# A P21-CRE MOUSE MODEL TO MONITOR AND MANIPULATE P21-HIGHLY-EXPRESSING SENESCENT CELLS IN VIVO

**DOI:** 10.1093/geroni/igad104.1358

**Published:** 2023-12-21

**Authors:** Ming Xu

**Affiliations:** UConn Health, Farmington, Connecticut, United States

## Abstract

The role of senescent cells has been implicated in various tissue dysfunctions associated with aging, obesity and other pathological conditions. We generated a p21-Cre mouse model, containing a p21 promoter-driving inducible Cre, enabling us to examine p21Cip1-highly expressing (p21high) cells, a previously unexplored cell population exhibiting several characteristics typical of senescent cells. By crossing p21-Cre mice with different floxed mice, we managed to monitor, sort, image, eliminate or modulate p21high cells in vivo. We showed that p21high cells can be induced by various conditions, and percentages of p21high cells varied from 1.5% to 10% across different tissues in 23-month-old mice. Intermittent clearance of p21high cells improved physical function in 23-month-old mice. p21-Cre mouse model might be a valuable and powerful tool for studying p21high cells to further understand the biology of senescent cells in aging.

